# LINE-1 Epigenetic Repression and Regulation of Immunity

**DOI:** 10.3390/v18080895

**Published:** 2026-08-14

**Authors:** Holly Jefferson, Helen M. Rowe

**Affiliations:** Centre of Immunobiology and Infection, Blizard Institute, Queen Mary University of London, London E1 2AT, UK; h.jefferson@qmul.ac.uk

**Keywords:** LINE-1, transposable elements, epigenetic repression, adaptive immunity, innate immunity, chimeric transcripts

## Abstract

The long interspersed element-1 (LINE-1) family is the sole remaining transposable element family that is capable of autonomous retrotransposition in humans. LINE-1 expression, tightly controlled through epigenetic mechanisms, has complex effects on gene regulation. Due to their similarity to retroviruses, once activated, LINE-1s have the ability to trigger pathways involved in innate and adaptive immunity with both beneficial and detrimental effects. This review will explore the mechanisms and importance of LINE-1 epigenetic repression and the potential outcomes of LINE-1 expression, with a focus on their immunogenic roles in innate and adaptive immunity.

## 1. Introduction

Since their first discovery in the 1940s by Barbara McClintock [1], transposable elements (TEs) have become a widely researched field. These mobile genetic elements, viral in origin, have integrated into the genome over millions of years through insertion into the germ line enabling TEs to amplify and remain stable in the genome [2,3]. Consequently, TEs make up almost half of the human genome [4,5].

There are two classes of TEs historically based on their method of transposition or ‘jumping’ [6]: DNA transposons, which act via a ‘cut-and-paste’ mechanism; and RNA retrotransposons that utilise an RNA intermediate in a ‘copy-and-paste’ mechanism, thus generating new copies in the genome and preserving the original copy. However, the majority of TEs have lost their ability to jump now due to truncations or mutations gained over millions of years, and negative selection based on their potential deleterious effects. Those younger TEs that have more recently integrated into the genome have not yet had the time to decay and as such are still mobile. The only remaining TEs that have retained their ability to jump autonomously in humans are the non-LTR RNA retrotransposons, long interspersed elements-1s (LINE-1s), which are part of the LINE family. Whilst there are roughly half a million LINE-1 copies present in the human genome occupying around 17% of our genome [5], only around 100 copies are still retrotransposition-competent [7]. LINE-1s can also mobilise non-autonomous TEs such as SVAs and Alu elements that do not contain their own machinery for retrotransposition [8,9].

**Figure 1:**
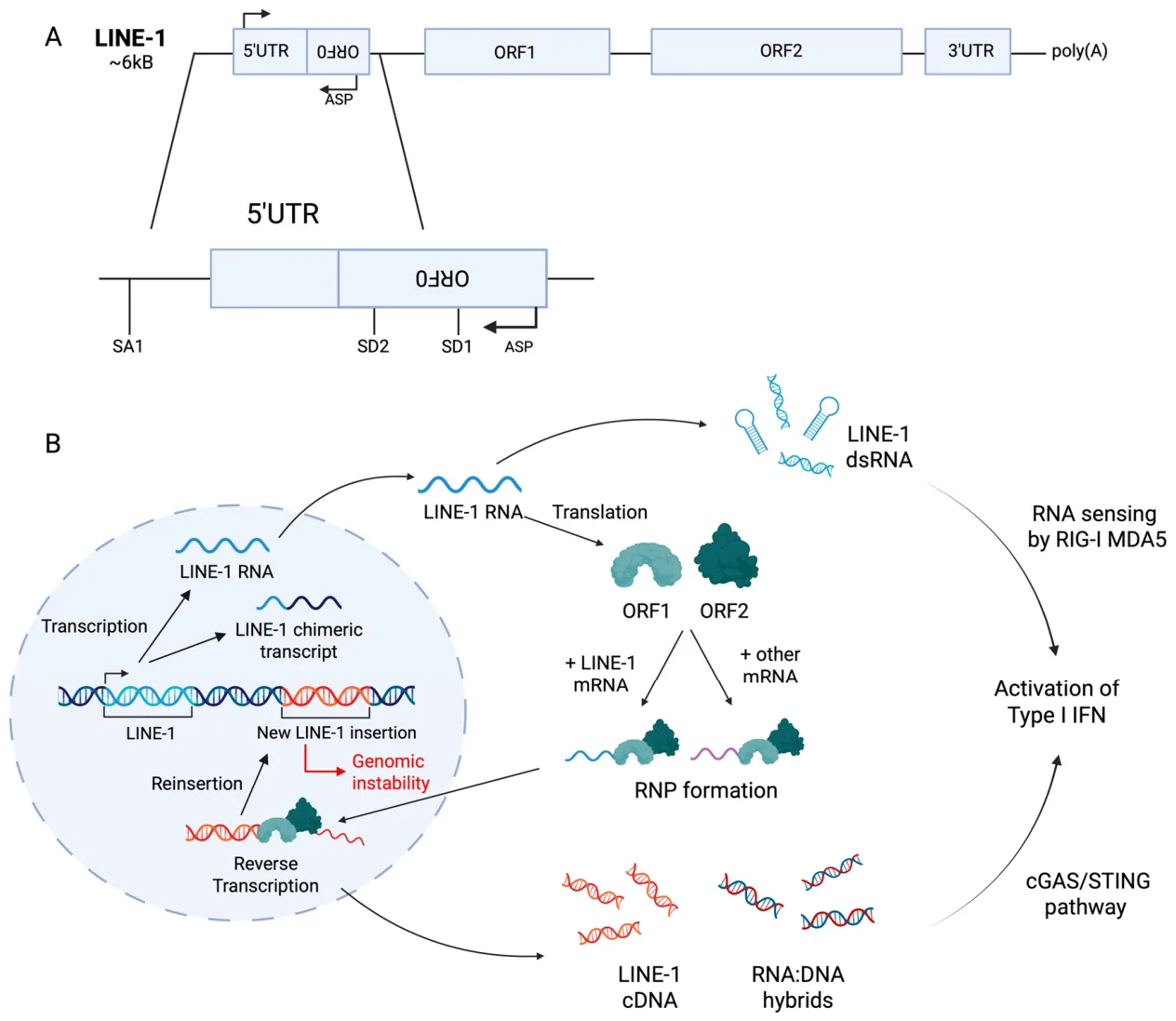
LINE-1 structure and life cycle. (**A**) LINE-1s are ~6 kb in length and are composed of a 5′UTR containing a sense Pol II promoter for two open reading frames (ORFs) ORF1 and ORF2, a 3′UTR and a poly(A) site. The 5′UTR also contains an antisense promoter (ASP) that drives expression of a third ORF, ORF0, in the antisense strand. Within ORF0 are two splice donor sites (SD1 and 2), which can splice into splice acceptor sites (SA1) that occur in nearby cellular genes downstream of ORF0 [24]. (**B**) LINE-1 life cycle [16]: Upon expression, LINE-1 RNA is produced as well as LINE-1 chimeric RNAs containing juxtaposed sequences of nearby genes. LINE-1 RNA is translated into ORF1 and ORF2 proteins which combine with LINE-1 RNA or other TE RNAs such as SINEs, to form the ribonuclear protein (RNP) complex. This re-enters the nucleus, and LINE-1 RNA will be reverse-transcribed and inserted into the DNA at a new site, driven by ORF2 activity. LINE-1 RNAs can also self-associate to form inverted-repeat RNAs or sense/antisense complementary dsRNAs that are sensed by the RNA sensor MDA5. LINE-1 RNAs can also activate RIG-I if they are transcribed from nearby Pol III promoters and contain a 5′-triphosphate. RNA sensing through RIG-I or MDA5, leads to activation of type I IFN signalling. LINE-1 RNA:DNA hybrids and cDNAs are further replication intermediates that can be shed and activate the TLR3 or cGAS/STING pathway, resulting in an IFN-I response. RNA:DNA hybrids can be generated through LINE-1 particles in the cytoplasm outside of TPRT due to LINE-1 overexpression.

LINE-1s are six kilobases in length (Figure 1A) and contain a 5′UTR, and two open reading frames (ORFs) required for LINE-1 retrotransposition (Figure 1B) under the control of a sense promoter. ORF1 encodes an RNA binding protein (ORF1p) [10], and ORF2 encodes reverse transcriptase and endonuclease activity (ORF2p) [11,12]. During translation of human LINE-1 mRNA, the ribosome terminates after ORF1 and reinitiates at ORF2, and this is thought to be driven by the RNA structure [13]. After translation, ORF1p and ORF2p combine with LINE-1 RNA or other TE RNA [8] to form ribonuclear protein (RNP) complexes [14]. In the nucleus, reverse transcription occurs by target-primed reverse transcription (TPRT), during which ORF2p nicks the DNA and uses this as a primer for cDNA synthesis [15,16,17,18]. ORF2p has recently been shown to contain binding sites for PCNA and PABPC1, which proposes a model whereby the interaction between ORF2p and PCNA could explain how ORF2p recruits the RNP to the target DNA for LINE-1 insertion in TPRT. However this role of PCNA has not been fully established [19]. Alternatively, in mouse LINE-1s upstream of each ORF, there are internal ribosomal entry sites (IRES) for recruiting ribosomes as another mechanism for LINE-1 translation [20]. A third ORF is located in the LINE-1 5′UTR, ORF0 [21,22], which is under the control of an antisense promoter (ASP) [23]. There is also some evidence that ORF0 can aid in LINE-1 mobility [24]; however, there is much more to explore regarding ORF0’s function.

Upon LINE-1 expression, the activated sense or antisense promoters of LINE-1s can also produce chimeric mRNAs through alternative splicing into exons of surrounding genes [23,25,26] and capturing polyadenylation signals. LINE-1s contain multiple splice donor (SD) sites in both sense and antisense strands which splice to splice acceptor (SA) sites in upstream genes [24,27]. The result is chimeric RNA transcripts containing sequences of both the LINE-1 and surrounding genes [28,29]. The antisense promoter drives transcription of ORF0, containing two splice donor sites, enabling formation of ORF0 chimeric transcripts which contain part of the 5′UTR of LINE-1s [24,30]. These commonly arise from younger LINE-1 subfamilies, L1PA1 to L1PA8 [31], and are often found upon antisense promoter hypomethylation [32,33]. LINE-1 associated transcripts are also generated through LINE-1 retrotransposition, whereby bystander cytoplasmic RNAs and chimeric RNAs can associate with RNPs and become reverse-transcribed by TPRT, before inserting into the genome at a new location as processed pseudogenes [34,35].

LINE-1s can influence gene expression by providing transcription factor-binding sites, and genes containing LINE-1s can become under the control of the LINE-1 sense or antisense promoters. Therefore, LINE-1 transcription influences the surrounding genes whereby silencing or activating LINE-1s has a corresponding effect on the nearby genes [36]. Subsequently, LINE-1-mediated gene expression can lead to new isoforms of gene products and can potentiate genome evolution [37]. In some cases, LINE-1s have been co-opted and selected for if the LINE-1 has a beneficial role to the host [38,39]. Examples of where LINEs may have beneficial roles include a L1M in the first intron of IFNAR that may potentially be co-opted to be activated by IFN to drive IFNAR expression [40], and a LINE-2 in the *CD274* gene that encodes PDL1, which when retained in the transcript can provide receptor antagonist activity to PDL1 [41]. LINE-1 insertion can also cause genomic instability due to the gene disruption and DNA damage caused, often with negative outcomes. Accordingly, LINE-1s and other TE insertions have been implicated in a plethora of diseases ranging from autoimmune diseases to neurodegenerative disorders [42,43]; thus, they are tightly regulated.

Due to the similarity of the LINE-1 replication cycle to that of retroviruses, with the difference that reverse transcription of RNA occurs in the nucleus for LINE-1s rather than in the cytoplasm as is the case for retroviruses such as HIV [44], LINE-1s produce nucleic acids as part of their life cycle which can act as danger signals to initiate innate immunity. This is called viral mimicry, whereby TEs can be viewed as ‘non-self’ by the host. Therefore, they are capable of inducing type I IFN (IFN-I) signalling cascades via RNA or DNA sensing in a similar fashion to invading viruses, implicating LINE-1s as culprits in triggering immunological responses. Notably though, LINE-1s can also generate short RNA:DNA hybrid products in the cytoplasm when overexpressed due to the accumulation of LINE-1 particles and the activity of their reverse transcriptase. Dependant on the circumstances under which LINE-1s are expressed, this can be either beneficial or detrimental.

This review will focus on epigenetic regulation of LINE-1s and their connection with innate and adaptive immunity, exploring the role of nucleic acid sensing and LINE-1 chimeric proteins, and whilst other TE families can have the same involvement (see [3]), they are beyond the scope of this review. In addition, important non-epigenetic mechanisms for LINE-1 regulation that occur throughout the LINE-1 life cycle are in place, and whilst these are not explored here, they are comprehensively detailed in these reviews [45,46,47].

## 2. LINE-1 Epigenetic Regulation

**Figure 2:**
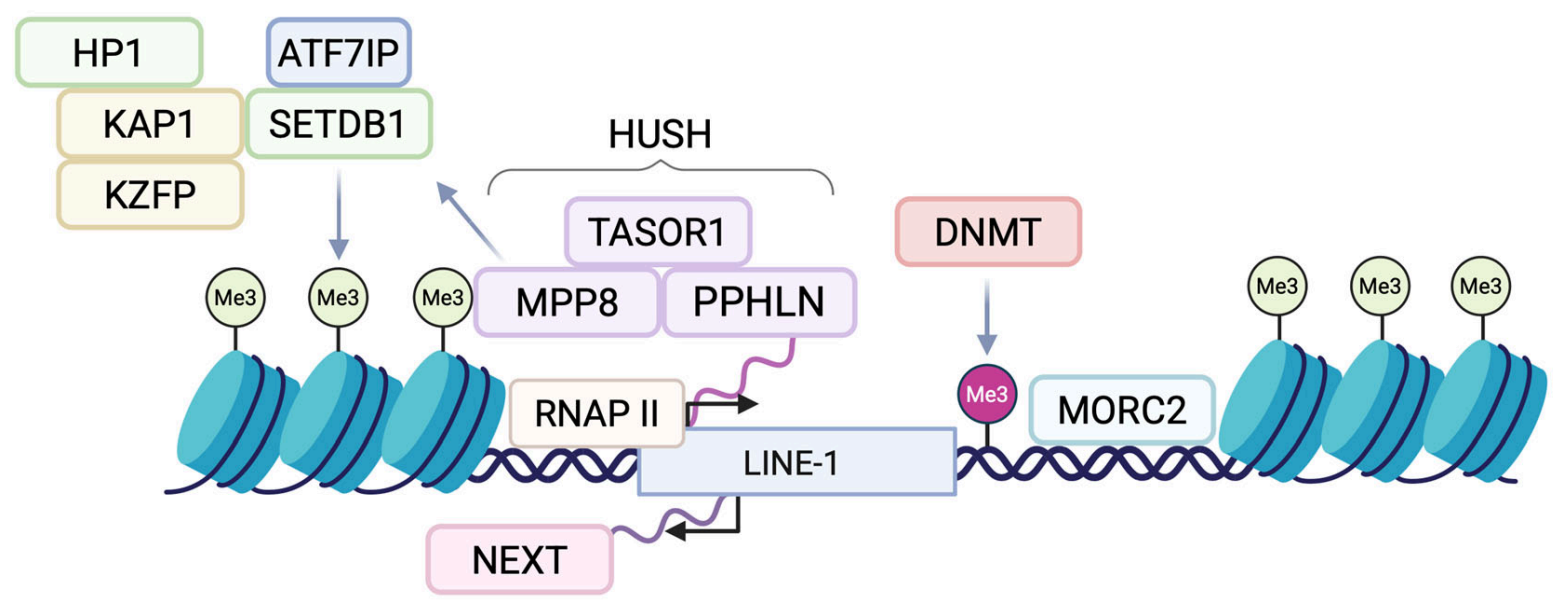
LINE-1 epigenetic regulation. LINE-1s are under the control of multiple epigenetic repressive mechanisms. The human silencing hub (HUSH) is composed of MPP8, periphilin (PPHLN), and TASOR1 [75]. MPP8 binds to H3K9me3 (Me3) and PPHLN binds to A-rich intronless LINE-1 RNAs that are being transcribed by RNA polymerase II (RNAP II) [78,81]. HUSH then drives chromatin compaction through the recruitment of SETDB1 [58], DNA methyltransferases (DNMTs) [54] and the MORC2 DNA-binding and compaction protein by MPP8 [78,85]. Additionally, the NEXT complex is recruited by HUSH to drive exosomal decay of non-polyadenylated LINE-1 antisense RNAs [88]. LINE-1s are also under the control of KRAB-ZNF-KAP1-mediated silencing, involving sequence-specific KRAB-ZNF proteins (KZFP) and their co-repressor KAP1 [50], recruiting histone methyltransferases (HMTs) and binding proteins, SETDB1, ATF7IP, HDACs, HP1 and DNMTs, acting as a hub for maintaining heterochromatin for LINE-1 repression [72].

LINE-1s have been implicated in a number of autoimmune diseases and cancer [48] due to the collateral damage that LINE-1 de-repression can have, including in driving inflammation. Accordingly, a range of mechanisms are in place for LINE-1 regulation. In particular, the focus of this review is the epigenetic regulatory pathways responsible for LINE-1 transcriptional silencing involving a host of proteins that modify the chromatin state at LINE-1 integrants (Figure 2) [49,50]. These epigenetic marks are established early in development and can be heritable in adult tissues [51,52]. The epigenetic repression of LINE-1s is primarily driven through chromatin compaction by controlling the methylation state of both DNA and histones, particularly at the LINE-1 promoter. Efficient control of LINE-1 expression is ensured through a network of chromatin-interacting proteins that spread heterochromatin locally.

LINE-1 DNA methylation and subsequent repression is the result of DNA methyltransferases (DNMTs) adding methyl groups onto CpG sites primarily at LINE-1 promoters, to produce heterochromatin and recruit additional chromatin remodelling proteins. There are three main DMNTs in humans responsible for DNA methylation, DNMT1, DNMT3A and DNMT3B [53], and their activity depends on their specific expression patterns through development and on the pre-existing methylation status of the DNA. DNMT3A and DNMT3B plus their cofactor DNMT3L are responsible for de novo methylation in early development, whereas DNMT1 has a preference for hemimethylated promoters and maintains DNA methylation during cell divisions and in somatic cells [54]. Similarly in mice, DNMT3C is active in the male germ cells, where it is responsible for controlling TE activity [55]. DNMTs can maintain LINE-1 repression if other forms of epigenetic regulation are lost [51,56], and even though it has been shown that sole loss of DNA methylation is not sufficient to fully activate LINE-1 expression [36], it remains the dominant method for LINE-1 epigenetic repression [57].

Histone methylation by histone methyltransferases (HMTs) can also induce chromatin compaction, often working in tandem with DNA methylation. An important HMT for repression of LINE-1s and other TEs is SETDB1 [58,59,60,61] that is responsible for histone H3K9me3 modifications, which silences retroelements in both embryonic stem cells [62,63] and in differentiated cells in adult tissues [50,64,65]. SETDB1 is recruited to TEs by KAP1 (also known as TRIM28), a co-repressor for KRAB zinc-finger proteins (KZFPs), although SETDB1 can silence LINE-1s both with and without KAP1 [65].

KAP1 also binds heterochromatin protein 1 (HP1) for histone methylation, and an inability for KAP1 to bind to HP1 results in a loss of heterochromatin and increased LINE-1 expression [50,66]. KAP1 is recruited to its targets by KRAB-ZNFs, of which there are hundreds in the genome that have co-evolved to match the TEs that have entered our genome, and are sequence-specific to a particular TE family [67,68]. In the case of LINE-1s, one such ZNF is ZNF93 that is responsible for the repression of older LINE-1 families (more than 15 million years old), often those that have lost the ability to retrotranspose [69]. KRAB-ZNFs are in an evolutionary arms race with LINE-1 elements and new subfamilies of LINE-1s can evolve following deletion of key sequences needed for ZFP binding. A 129bp deletion in the LINE-1 5′UTR led to escape from ZNF93 repression, for example, with younger LINE-1 subfamilies (L1HS and L1PA2) lacking this sequence [68]. ZNF695 also regulates LINE-1 retrotransposition, through DNA binding and recruitment of DNMTs for LINE-1 methylation [70].

Additional HMTs interacting with H3K9me3 marks are Suv39H and GPa/GLP, which have been shown to maintain histone methylation at LINE-1s [71]. These HMTs interact together with SETDB1, forming a multimeric complex for effective H3K9 methylation [72]. GPa/GLP can also methylate other non-histone proteins, including ATF7IP which can recruit further chromatin modifiers, including SETDB1 and MPP8, as well as protecting SETDB1 from proteasomal degradation [73,74]. The loss of any of these HMTs can prevent heterochromatin formation and LINE-1 repression.

The human silencing hub (HUSH) has also been implicated in LINE-1 regulation. First discovered as an epigenetic silencer through a genome-wide screen into position-effect variegation [75], we and others have since shown that HUSH regulates LINE-1 expression [76,77] through binding preferentially to LINE-1 integrants and maintaining their transcriptional repression [78,79]. The HUSH complex is composed of three proteins, MPP8, TASOR (FAM208A), and periphilin, and silences active LINE-1s [80]. TASOR binds to MPP8 and periphilin to form the complex and localises HUSH to H3K9me3 on LINE-1s via MPP8 with its pseudo-PARP domain [81]. Periphilin binds to LINE-1 RNA actively being transcribed by RNA polymerase II (RNAP II) [78,82,83], and MPP8’s chromodomain will bind to the H3K9me3 and recruit accessory proteins for chromatin compaction, including SETDB1 [84], DNMT3A, and MORC2, an ATPase [85] that binds directly to DNA, and upon ATP hydrolysis will promote chromatin compaction [86,87]. HUSH also recruits the nuclear exosome targeting (NEXT) complex, composed of ZCCHC8, MTR4 and RBM7, to drive exosome-mediated degradation of LINE-1 RNA [88]. However, HUSH can also mediate H3K9me3-independent silencing, whereby HUSH targets sites with high RNAP II levels, transcribed exons and transcriptional start sites, instead of those with H3K9me3 marks such as LINE-1s. This ensures effective silencing of non-self-targets and increases the adaptability of HUSH silencing [89].

The recently discovered second HUSH complex, HUSH2, is also involved in regulation of innate immunity [90,91]. Whilst still composed of MPP8 and periphilin, it instead contains a paralog of TASOR, TASOR2, that competes with TASOR for its remaining subunits. Rather than H3K9me3 sites, HUSH2 is targeted to H3K4me3 found on promoters of interferon stimulated genes (ISGs) to silence ISG expression. The expression and activity of HUSH1 and HUSH2 complexes are increased in early development in human pluripotent cells to prevent unwanted ISG activation through dual LINE-1 and ISG promoter repression [92].

The method of epigenetic regulation is often dependent on the age of the LINE-1. The older families, L1PA3 to L1PA8, are subject to KAP1-mediated silencing via KZFPs, whereas the younger L1HS to L1PA3 families are primarily regulated through DNA and histone methylation and the HUSH complex. LINE-1 repression is also often time-specific, as MPP8 has been shown to be responsible for maintaining pluripotency of mESCs through LINE-1 repression [84], and some LINE-1 RNAs are actively expressed in human iPSCs which guide neural differentiation [36,93].

Stringent epigenetic repressive mechanisms of LINE-1s are vital, and an overlap between HUSH-mediated silencing and DNA and histone methylation enables effective LINE-1 repression. MPP8 mediates the interaction of GLP/G9a and Dnmt3a, the mouse DNMT3A [94], and there is an overlap between genes repressed by HUSH and SETDB1/KAP1 [77]. The interaction between chromatin-associated readers and writers ensures a multilayered approach to LINE-1 silencing.

### LINE-1 Epigenetic Dysregulation

In certain permissive cell states, epigenetic silencing breaks down, leading to LINE-1 transcription. For example, in cellular senescence, LINE-1 becomes de-repressed through altering expression of factors such as RB1 and FOXA1. Downregulation of RB1 results in loss of heterochromatin at LINE-1s, and upregulation of FOXA1 will activate the LINE-1 promoter. The resulting induction of type I IFN signalling due to LINE-1 expression contributes to maintaining a senescent state [95]. Transcription factor binding can also enhance LINE-1 activity to promote cellular senescence; for example, PAX5 binds to the LINE1 5’UTR resulting in activation for senescence [96].

Epigenetic dysregulation has been observed in the context of some diseases such as cancer or autoimmune disease, although not in all cases. The loss of the HUSH complex due to downregulation of MPP8 has been shown to occur in some cancer patients and is accompanied with an induction of type I IFN [80]. There is also a correlation between LINE-1 expression and genome-wide hypomethylation, and a loss of DNMTs results in increased LINE-1 expression. Thus, LINE-1 hypomethylation can be a hallmark of cancer [97], highlighting the potential to use LINE-1 methylation status as a therapeutic biomarker for cancer [98,99].

LINE-1s can also be regulated by controlling their RNA levels. In addition to HUSH regulation of LINE-1 RNA tethered to chromatin due to periphilin’s RNA binding activity [82,83], PIWI-interacting RNAs (piRNAs) also target full-length LINE-1 RNA in humans through promoting LINE-1 DNA and H3K9me3 marks, increasing epigenetic repressive marks and chromatin compaction [100]. LINE-1 RNA that reaches the cytoplasm is regulated by ISG-derived proteins, such as MOV10 and HELZ2, and other host innate immune mechanisms [101,102] which are detailed later in this review. Furthermore, through additional genome-wide screens, several other regulators of LINE-1 expression have been identified [63,103,104,105], but their role in LINE-1 repression is not further explored in this review.

## 3. LINE-1s and Innate Immunity

Upon viral invasion, cells utilise sensing pathways to recognise viral DNA and RNA via pattern recognition receptors, to induce inflammation and type I IFN signalling for antiviral innate immunity. Type I IFNs, primarily IFNα and IFNβ, bind to the interferon-α/β receptor (assembled from IFNAR1 and 2 gene products) activating JAK-STAT signalling [106]. Active phosphorylated STAT1 and 2 form the interferon-stimulated gene factor 3 (ISGF3) complex with interferon regulatory factor 9 (IRF9). This then migrates to the nucleus to initiate transcription of hundreds of IFN-stimulated genes (ISGs) that are regulated by the IFN-stimulated response element (ISRE) [107], enabling the host to mount an effective response to the invading virus. However, expression of transposable elements, including LINE-1s and Alus can also produce nucleic acids that can be recognised as ‘non-self’ and activate the same sensing pathways as viruses, referred to as viral mimicry. These LINE-1 and Alu-derived nucleic acids can initiate a type I IFN response that may prime or re-enforce innate immunity [108,109].

LINE-1s can activate the RNA sensing pathway [80,110] through several mechanisms involving dsRNA-recognition by RIG-I-like receptors (RLRs), RIG-I and MDA5 [111,112,113]. Upon LINE-1 dsRNA binding, RIG-I and MDA5 activate MAVS adaptor protein that subsequentially mediates a downstream signalling cascade to activate TBK1, which phosphorylates and activates IRF3 and 7 [114,115,116]. In a similar process, LINE-1 cDNAs can activate type I IFN signalling via the DNA sensor cGAS (cyclic GMP-AMP synthase). Despite the ability of cGAS to sense fragments as small as 40bp, the larger the DNA fragment the larger the IFN response [117]. Upon sensing of LINE-1 cDNAs, cGAS produces cGAMP that binds to and activates stimulator of IFN genes (STING), which also recruits TBK1 and IKKε kinases for NF-κB production and IRF3 activation [109,118,119]. The end result of both RNA and DNA sensing pathways is the transcription of type I IFNs, which are secreted and taken up by the type I IFN receptor, leading to activation of ISGs through JAK-STAT signalling. In parallel, dsRNA sensing also leads to the activation of NF-κB as well as NF-κB target genes [120]. As mentioned above, RNA:DNA hybrids generated by ORF2 reverse-transcriptase in the cytoplasm (and independent of TPRT [12]) also induce the cGAS-STING signalling pathway [121] and additionally activate toll-like receptor 3 (TLR3) to potentiate the RLR sensing pathway [110].

Alu elements, whilst an independent class of TEs, can be mobilised by LINE-1s and are well-documented to trigger innate immune responses and autoinflammation [122]. Alus are a type of short interspersed nuclear elements (SINEs) that rely on LINE-1 for their retrotransposition and constitute 10% of the human genome [5]. Similar to LINE-1s, when two Alus are present in opposite orientations within the same gene, an RNA transcript can fold on itself to form an inverted Alu repeat (IR-Alus) dsRNA [123]. These IR-Alus activate MDA5 and RIG-I, potentiating a downstream response leading to ISG and IFN induction [124,125]. Protein kinase R (PKR) is also activated by IR-Alus triggering global translational shut down and apoptotic cell death, thus inducing IR-Alu expression is a promising avenue to promote PKR-driven apoptosis for disease treatments [126,127]. Endogenous retroviruses (ERVs) also have a plethora of roles in innate immunity but are not discussed in this review.

Chronic type I IFNs associated with LINE-1s and Alu nucleic acids have many negative downstream consequences [123] and are often implicated in many autoimmune and autoinflammatory diseases, including Aicardi–Goutières syndrome (AGS) and systemic lupus erythematosus (SLE) [128]. In AGS, loss-of-function mutations in ADAR1, a dsRNA editing protein, and gain-of-function mutations in MDA5, such as M854K MDA5 that has a stronger binding affinity to Alu dsRNAs through inhibition of ATP hydrolysis, result in the constitutive activation of MDA5 and IFN-I signalling causing chronic inflammation [124,129,130,131]. Similarly, a loss of TREX1, a DNA exonuclease responsible for degrading cytosolic DNA, results in a build-up of LINE-1 cDNA and chronic cGAS-STING signalling [132]. High cGAS-STING activity in senescent cells also promotes inflammation associated with ageing and neurodegeneration [95,133].

Early in human development, embryos are preserved in an immune-privileged state, and so to protect embryos from viruses and endogenous dsRNAs such as those arising from retroelements, there is upregulation of all HUSH complexes through increased expression of MPP8. This results in increased suppression of LINE-1 elements and ISGs directly through HUSH1/2 to ensure that embryos are protected from lethal IFN activation [92]. Additionally, it has been shown in mouse embryonic stem cells (mESCs) that dsRNA recognition is inhibited by repressing MDA5 and subsequently the RLR RNA sensing pathway [134]. An IFN response in these contexts would otherwise disrupt stem cell differentiation and development [92,134].

LINE-1 nucleic acid sensing has also been shown to have beneficial effects in certain contexts. An upregulation of LINE-1s was observed in fractured bones in mice, and inflammation upon LINE-1 dsRNA transfection into osteoblasts induces bone mineralisation and repair [135].

Cells have a variety of innate mechanisms to defend themselves against TE nucleic acid sensing, and type I IFNs induced by TE nucleic acids can have a negative feedback effect on LINE-1 expression. This can be via direct LINE-1 repression: for example, upon DNA damage induced due to LINE-1 retrotransposition cGAS becomes nuclear and interacts with the E3 ligase TRIM41 to repress LINE-1s via ubiquitination and degradation of ORF2 protein [136]. Similarly, activated MDA5 can bind to the LINE-1 5′UTR and ADAR1 can bind to the LINE-1 RNP complex to suppress retrotransposition [137,138]. Alternatively, the TE nucleic acids themselves can be degraded to prevent a type I IFN response. TREX1 negatively regulates cGAS-STING by cleaving the nucleic acids [109,139] and DGCR8, a dsRNA binding protein, binding to LINE-1 dsRNAs to prevent its accumulation to inhibit the downstream signalling of the MDA5-MAVS pathway [140]. In addition, various ISGs expressed as the result of IFN signalling have been shown to repress LINE-1 activity, including the helicase MOV10 and RNASEL that inhibit retrotransposition by restricting LINE-1 mobility through aggregation into stress granules and LINE-1 RNA degradation, respectively [101,141,142,143].

However, it has been suggested that LINE-1 activation can counteract the negative feedback of innate immune responses. In pancreatic adenocarcinoma, LINE-1 ORF1p may have a protective role through localisation with MOV10 to shield dsRNAs from RIG-I/MDA5 sensing and IFN signalling to evade LINE-1 silencing, which could contribute to pancreatic adenocarcinoma tumour growth. However, it remains unclear whether this is a common immune evasion strategy that might operate in other cancers [144]. Other examples where disrupting nucleic acid sensing pathways has the potential to promote cancer development include DNA tumour viruses, such as HPV and adenovirus that have specific oncogenes, E7 and E1A respectively, which bind to STING to inhibit the cGAS-STING pathway [145]. In TP53 mutated pancreatic ductal adenocarcinoma there is increased LINE-1 retrotransposition, and ORF1p can negatively regulate cGAS-STING by binding to TE nucleic acids to prevent sensing [146]. Furthermore, in some cancers such as acute myeloid leukaemia, SETDB1 is overexpressed to silence TE expression and the corresponding dsRNAs, to prevent the IFN response which could negatively affect cancer survival [60]. Consequently, the interaction between TEs and innate immune receptors provides many promising targets to pursue in new cancer therapies [147], as inhibiting repressors of LINE-1 has downstream immune consequences (Table 1). Stimulating nucleic acid sensing pathways using ADAR1 inhibitors enhances IR-Alu sensing [148]. DNA methylation inhibitors, such as 5-AZA, are widely used as cancer treatments to unleash a cytoplasmic influx of IR-Alus to induce an IFN response, resulting in reduced proliferation of colorectal cancer-initiating cells, increased apoptosis and a response of melanomas to immune checkpoint therapies [115,116,148].

**Table 1:** Repressors of LINE-1, their targets and the consequence of de-repression of these repressors.

Repressors	Targets	Immune Consequences of De-Repression	References
HUSH	LINE-1 DNA and RNA (on chromatin)	Nucleic acid sensing, IFN signalling, activation of retrotransposition.	[76,77,80,90]
ADAR1	LINE-1 dsRNA and RNP	MDA5 sensing and IFN signalling.	[129,148]
MOV10 HELZ2 DGCR8	LINE-1 RNA/dsRNA	RIG-I/MDA5 sensing and IFN signalling	[101,141,142] [102] [140]
TREX1	LINE-1 cDNA	cGAS/STING activation and IFN signalling	[132,139]

## 4. LINE-1s and Adaptive Immunity

In addition to triggering IFN responses for innate immunity, LINE-1s can also elicit an adaptive immune response, due to the capability of LINE-1s and other TEs to encode proteins or chimeric proteins that can serve as antigens to stimulate T cells. Generally, this should not occur in healthy cells due to epigenetic silencing; therefore, most information regarding LINE-1s and adaptive immune responses has been uncovered in the context of cancer and autoimmune diseases. Despite this, recent discoveries have found that a subset of LINEs, along with other TEs, could be found expressed in both mouse and human thymus, which is responsible for T cell maturation and negative selection of self-reactive T cells [149,150,151]. Exactly which LINE-1-chimeric peptides may be viewed as self vs. foreign depend on their expression in the thymus, and how this impacts LINE-1-specific T cells remains to be determined.

It has also been suggested that LINE-1 expression could be implicated in modulating T cell activity. To mount an effective response, adult T cells must remain quiescent and primed ready for proliferation and differentiation [152]. Non-canonical LINE-1 transcripts, particularly those from the L1M family, have the potential to enforce naïve CD4+ T cell quiescence by associating with active chromatin. Upon T cell activation, mTORC signalling can drive suppression of non-canonical LINE-1 splicing through PTBP1, which could permit expression of genes involved in the T cell activation programme [153]. Conversely, neonatal naïve T cells lack LINE-1 expression due to mTORC1- and PTBP-1-mediated suppression of LINE-1 splicing, enabling protein synthesis and priming for T cell activation. However, LINE-1 expression can rise progressively with age, suppressing protein synthesis and contributing to T cell exhaustion and immune senescence [154]. In addition, upon damage to the skin barrier, LINE-1s have been described to be upregulated in the antigen presenting Langerhan cells, where four peptides derived from LINE-1s were detected. These trigger IL-17A and TNFα production and activate LINE-1-specific CD8+ T cells, aiding in wound healing and tissue regeneration in the skin [155]. However, there are cases where LINE-1-mediated T cell activity is detrimental. LINE-1 hypomethylation has been identified as a hallmark of Coeliac disease with a predisposition to bowel adenocarcinomas, a disease characterised by aberrant T cell activity. This was shown to result in an upregulation of T cell activity as well as genes associated with inflammation and the immune response. Whilst LINE-1 hypomethylation did not lead to ORF1p and ORF2p expression, it is possible that the inflammatory response could be caused by LINE-1 nucleic acid or RNA:DNA hybrid sensing, similarly shown in other autoimmune diseases. However, this is currently unknown [156].

Stimulation of the adaptive immune system by LINE-1s can also occur through their ability to form chimeric transcripts which, along with LINE-1 canonical transcripts and other TE transcripts, can be translated and subsequently processed to produce peptides. These are then able to be presented by MHC class I on the surface of cells due to their strong binding affinity for HLA molecules [97,157,158] and can serve as antigens to activate T cells to stimulate an adaptive immune response [159,160]. These chimeric proteins derived from chimeric transcripts additionally increase protein diversity. In cells overexpressing LINE-1s, peptides from ORF1p are detectable, both presented by MHC molecules on the cell surface and circulating in patient blood samples, and CD4+ and CD8+ T cells can be activated in response to the ORF1p peptides, alongside an increase in IFN-ɣ signalling [161]. In another study, healthy PBMCs were pulsed with TE chimeric peptides leading to activation and proliferation of CD8+ T cells akin to the host response to the Epstein-Barr virus [162].

Due to LINE-1 overexpression commonly being observed in cancer, the majority of investigations into LINE-1-derived antigens have been conducted in this context [161,163,164] where we typically see LINE-1 hypomethylation and expression of TE chimeric transcripts [30,97,165]. In pancancer studies, TE chimeric transcripts were detectable across cancers and can produce tumour-specific TE chimeric neoantigens [26,158,162]. A well-documented LINE-1 chimeric transcript, L1-Met, has been shown to be produced in a number of cancers and has the potential to be a used as a diagnostic marker [33,165,166].

Exploiting this knowledge regarding TE chimeric transcripts and their role in initiating the adaptive immune response, targeting chromatin regulators of TE expression can enhance susceptibility of cancer cells to immune checkpoint blockade therapies. In SETDB1-KO cancer cells, a number of loci containing TEs and immune-related genes involved in IFN-signalling and antigen presentation were expressed. The activation of these loci results in production of MHC class I peptides derived from TEs that can be recognised by CD8+ T cells to drive T cell cytotoxicity and an immune response against the tumour [65]. Furthermore, SETDB1 inactivation in tumours results in increased TE expression and response to anti-PDL1 therapies [167]. In glioblastoma and colorectal cancer, inhibiting DNMTs with 5-AZA, increases LINE-1 chimeric transcripts and presention of the corresponding peptides on MHC class I [97,168]. The loss of these epigenetic regulators results in TE activation and the production of TE tumour-specific antigens, which can stimulate an anti-tumour immune response [169].

**Figure 3:**
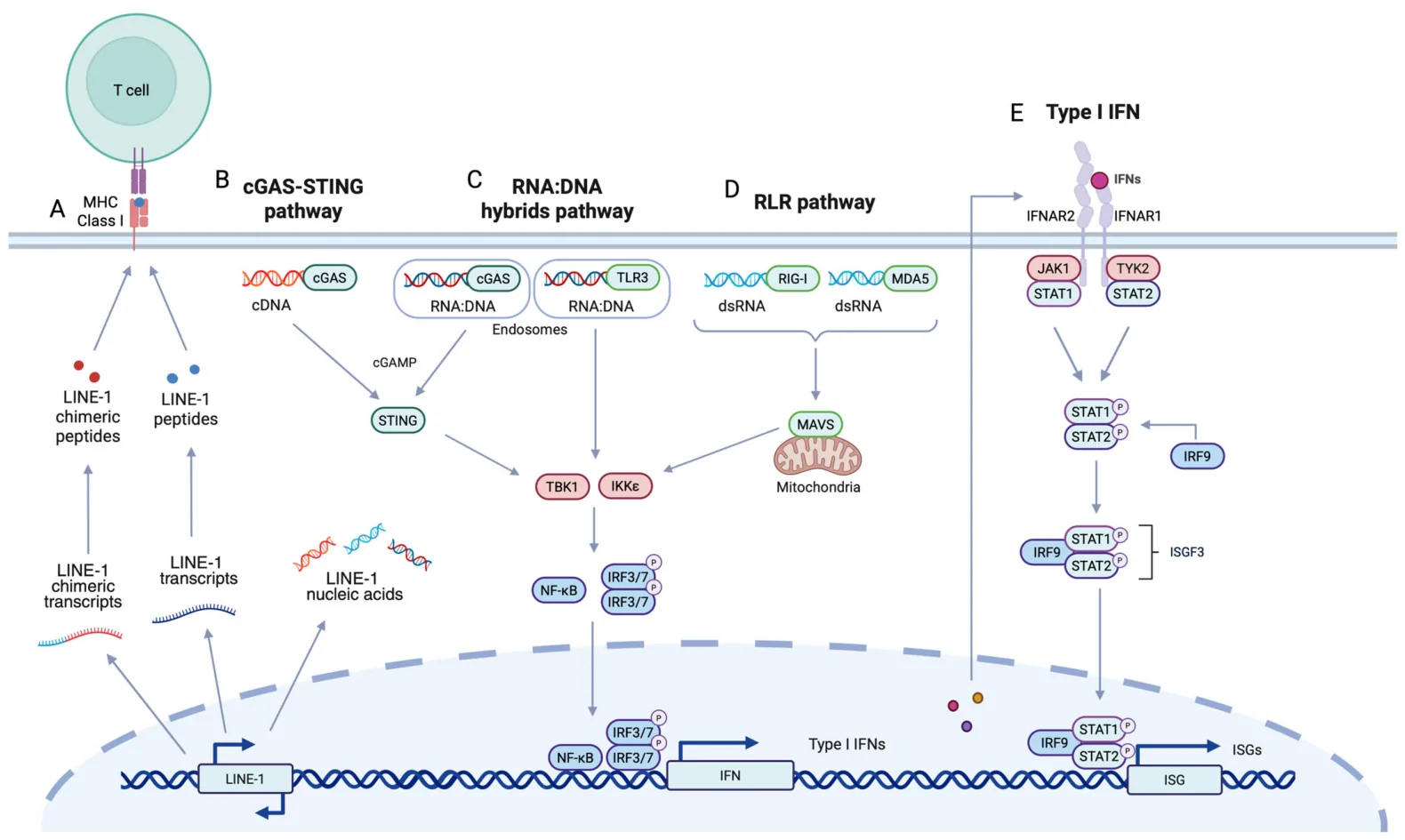
LINE-1 innate and adaptive immunity. (**A**) LINE-1 expression produces canonical and chimeric transcripts with nearby genes, which can be translated and processed into peptides that are then presented on MHC class I on the cell surface to stimulate T cells [158]. (**B**) LINE-1 cDNAs produced following LINE-1 reverse transcription activate the cGAS/STING pathway. cDNA binds to cGAS which produces cGAMP that binds to and activates STING. This will recruit the kinases TBK1 and IKKε to phosphorylate IRF3 and IRF7 and activate NFκB [120]. (**C**) RNA:DNA hybrids in endosomes can bind to either cGAS to activate the cGAS-STING pathway [121] or bind Toll-like receptor 3 (TLR) that subsequently dimerises. These pathways converge to also activate TBK1 and IKKε [110]. (**D**) LINE-1 and Alu dsRNA (either foldable IR-RNAs or sense/antisense annealed RNAs) will activate the RIG-I like receptor (RLR) RNA sensing pathway. dsRNAs bind to two RLRs, RIG-I (which recognises uncapped RNAs) and MDA5 (which recognises long dsRNAs), which activate MAVS found on mitochondria. Activated MAVS will recruit TBK1 and IKKε for IRF3/7 activation and NFκB production. IRF3/7 will dimerise, and along with NFκB, will bind to DNA and activate transcription of IFN-α/β genes for type I IFN production [111]. (**E**) Type I IFNs will be secreted and will then bind to their receptors IFNAR1 and 2, leading to receptor dimerization. Tyrosine phosphorylation by TYK2 and JAKs results in activation of JAK1, STAT1 and STAT2. STAT1/2 binds to IRF9 to form the ISGF3 complex, which enters the nucleus to bind to the interferon-stimulated response element (ISRE) to drive transcription of interferon-stimulated genes (ISGs) [106].

The repetitive nature and low mappability of LINE-1s and other TEs mean that identifying TE-chimeric transcripts by standard RNA-sequencing methods is limited. Therefore, in recent years a number of bioinformatics techniques have been developed, including long-read RNA sequencing (lr-RNA-seq), enabling unique mapping of TEs and the identification of isoforms and alternative transcripts. The development of lr-RNA seq has enabled further investigation into TEs and it is becoming the staple analytic technique for LINE-1 research. A number of studies have successfully used lr-RNA seq to study TE-chimeric transcripts and their corresponding antigens [160,164], and more focus has been on connecting TEs to adaptive immunity. Through combining lr-RNA seq with immunopeptidomics [158], the role of TE chimeric transcripts and their antigens in adaptive immunity is now being elucidated. However, there is much more research needed to fully uncover the role of LINE-1s in both innate and adaptive immunity (outlined in Figure 3), in both diseased and healthy states.

## 5. Conclusions

The outcome of LINE-1 expression is complex and can act in a variety of ways with negative consequences, though the effects can be beneficial in some circumstances. Whilst there are many well-defined methods of LINE-1 epigenetic repression, namely through chromatin readers and writers working in tandem to establish heterochromatin, including the HUSH complex, DNMTs, HMTs and KZNF-KAP1 that are explored in this review, there are many cases where there is disruption to these mechanisms. This is either intentional expression at specific stages of development and in co-option, or in disease settings such as autoimmune disease and cancer [48,170,171].

Here, we discuss the emerging role of LINE-1s in innate and adaptive immunity. Through the production of LINE-1 cDNAs and dsRNA, including inverted repeat Alus, viral mimicry is induced, which activates sensing pathways to initiate an IFN response akin to the expected host response to an invading virus. In addition, recent bioinformatic techniques have enabled the discovery of the processing of chimeric transcripts derived from TEs into peptides, with the capability to be presented on the cell surface on MHC molecules to act as epitopes that stimulate the adaptive immune response.

Further work, however, is required to fully elucidate the immunogenic role of LINE-1s and other transposable elements, in particular their role in adaptive immunity and the overlap between LINE-1- and HUSH2-mediated ISG responses that is unexplored in this review. This continually expanding area of research has the potential to reveal valuable insights regarding LINE-1 nucleic acids and their chimeric transcripts in disease contexts, and the scope that these provide can be utilised to develop therapies or act as biomarkers.

## Data Availability

No new data were created or analyzed in this study. Data sharing is not applicable to this article.

## References

[1] McClintock B. (1956). Controlling Elements and the Gene. Cold Spring Harb. Symp. Quant. Biol..

[2] Dewannieux M., Heidmann T. (2013). Endogenous Retroviruses: Acquisition, Amplification and Taming of Genome Invaders. Curr. Opin. Virol..

[3] Kassiotis G., Stoye J.P. (2016). Immune Responses to Endogenous Retroelements: Taking the Bad with the Good. Nat. Rev. Immunol..

[4] de Koning A.P.J., Gu W., Castoe T.A., Batzer M.A., Pollock D.D. (2011). Repetitive Elements May Comprise Over Two-Thirds of the Human Genome. PLoS Genet..

[5] Lander S., Linton L.M., Birren B., Nusbaum C., Zody M.C., Baldwin J., Devon K., Dewar K., Doyle M., FitzHugh W. (2001). Initial Sequencing and Analysis of the Human Genome International Human Genome Sequencing Consortium. Nature.

[6] Wicker T., Sabot F., Hua-Van A., Bennetzen J.L., Capy P., Chalhoub B., Flavell A., Leroy P., Morgante M., Panaud O. (2007). A Unified Classification System for Eukaryotic Transposable Elements. Nat. Rev. Genet..

[7] Brouha B., Schustak J., Badge R.M., Lutz-Prigge S., Farley A.H., Moran J.V., Kazazian H.H. (2023). Hot L1s Account for the Bulk of Retrotransposition in the Human Population. Proc. Natl. Acad. Sci. USA.

[8] Wei W., Gilbert N., Ooi S.L., Lawler J.F., Ostertag E.M., Kazazian H.H., Boeke J.D., Moran J.V. (2001). Human L1 Retrotransposition: CisPreference versus Trans Complementation. Mol. Cell. Biol..

[9] Esnault C., Maestre J., Heidmann T. (2000). Human LINE Retrotransposons Generate Processed Pseudogenes. Nat. Genet..

[10] Kolosha V.O., Martin S.L. (1997). In Vitro Properties of the First ORF Protein from Mouse LINE-1 Support Its Role in Ribonucleoprotein Particle Formation during Retrotransposition. Proc. Natl. Acad. Sci. USA.

[11] Feng Q., Moran J.V., Kazazian H.H., Boeke J.D. (1996). Human L1 Retrotransposon Encodes a Conserved Endonuclease Required for Retrotransposition. Cell.

[12] Baldwin E.T., van Eeuwen T., Hoyos D., Zalevsky A., Tchesnokov E.P., Sánchez R., Miller B.D., Di Stefano L.H., Ruiz F.X., Hancock M. (2023). Structures, Functions and Adaptations of the Human LINE-1 ORF2 Protein. Nature.

[13] Alisch R.S., Garcia-Perez J.L., Muotri A.R., Gage F.H., Moran J.V. (2006). Unconventional Translation of Mammalian LINE-1 Retrotransposons. Genes Dev..

[14] Doucet A.J., Hulme A.E., Sahinovic E., Kulpa D.A., Moldovan J.B., Kopera H.C., Athanikar J.N., Hasnaoui M., Bucheton A., Moran J.V. (2010). Characterization of LINE-1 Ribonucleoprotein Particles. PLoS Genet..

[15] Ostertag E.M., Kazazian H.H. (2001). Biology of mammalian L1 retrotransposons. Annu. Rev. Genet..

[16] Bergin C.J., Mendes da Silva A., Benoit Y.D. (2023). Where to Draw the LINE—Are Retrotransposable Elements Here to Stay?. Cancers.

[17] Miller I., Totrov M., Korotchkina L., Kazyulkin D.N., Gudkov A.V., Korolev S. (2021). Structural Dissection of Sequence Recognition and Catalytic Mechanism of Human LINE-1 Endonuclease. Nucleic Acids Res..

[18] Flasch D.A., Macia Á., Sánchez L., Ljungman M., Heras S.R., García-Pérez J.L., Wilson T.E., Moran J.V. (2019). Genome-Wide de Novo L1 Retrotransposition Connects Endonuclease Activity with Replication. Cell.

[19] Ghanim G.E., Hu H., Boulanger J., Nguyen T.H.D. (2025). Structural Mechanism of LINE-1 Target-Primed Reverse Transcription. Science.

[20] Li P.W.L., Li J., Timmerman S.L., Krushel L.A., Martin S.L. (2006). The Dicistronic RNA from the Mouse LINE-1 Retrotransposon Contains an Internal Ribosome Entry Site Upstream of Each ORF: Implications for Retrotransposition. Nucleic Acids Res..

[21] Boeke J.D., Fenyo D. (2015). Much Ado about Zero. Cell.

[22] Martin M.D., Brown D.N., Ramos K.S. (2021). Computational Modeling of RNase, Antisense ORF0 RNA, and Intracellular Compartmentation and Their Impact on the Life Cycle of the Line Retrotransposon. Comput. Struct. Biotechnol. J..

[23] Mätlik K., Redik K., Speek M. (2006). L1 Antisense Promoter Drives Tissue-Specific Transcription of Human Genes. BioMed Res. Int..

[24] Denli A.M., Narvaiza I., Kerman B.E., Pena M., Benner C., Marchetto M.C.N., Diedrich J.K., Aslanian A., Ma J., Moresco J.J. (2015). Primate-Specific ORF0 Contributes to Retrotransposon-Mediated Diversity. Cell.

[25] Kim S., Kim Y.J., Han K. (2016). Analysis of L1-Chimeric Transcripts Derived from Bidirectional Promoter of Human-Specific L1. Genes Genom..

[26] Clayton E.A., Rishishwar L., Huang T.C., Gulati S., Ban D., McDonald J.F., Jordan I.K. (2020). An Atlas of Transposable Element-Derived Alternative Splicing in Cancer. Philos. Trans. R. Soc. B Biol. Sci..

[27] Belancio V.P., Hedges D.J., Deininger P. (2006). LINE-1 RNA Splicing and Influences on Mammalian Gene Expression. Nucleic Acids Res..

[28] Nigumann P., Redik K., Mätlik K., Speek M. (2002). Many Human Genes Are Transcribed from the Antisense Promoter of L1 Retrotransposon. Genomics.

[29] Speek M. (2001). Antisense Promoter of Human L1 Retrotransposon Drives Transcription of Adjacent Cellular Genes. Mol. Cell. Biol..

[30] Cruickshanks H.A., Tufarelli C. (2009). Isolation of Cancer-Specific Chimeric Transcripts Induced by Hypomethylation of the LINE-1 Antisense Promoter. Genomics.

[31] Pinson M.E., Court F., Masson A., Renaud Y., Fantini A., Bacoeur-Ouzillou O., Barriere M., Pereira B., Guichet P.O., Chautard E. (2022). L1 Chimeric Transcripts Are Expressed in Healthy Brain and Their Deregulation in Glioma Follows That of Their Host Locus. Hum. Mol. Genet..

[32] Criscione S.W., Theodosakis N., Micevic G., Cornish T.C., Burns K.H., Neretti N., Rodić N. (2016). Genome-Wide Characterization of Human L1 Antisense Promoter-Driven Transcripts. BMC Genom..

[33] Cervantes-Ayalc A., Ruiz Esparza-Garrido R., Velázquez-Flores M.Á. (2020). Long Interspersed Nuclear Elements 1 (LINE1): The Chimeric Transcript L1-MET and Its Involvement in Cancer. Cancer Genet..

[34] Law C.-T., Burns K.H. (2026). Comparative Genomics Reveals LINE-1 Recombination with Diverse RNAs. Cell Genom..

[35] Buzdin A., Gogvadze E., Kovalskaya E., Volchkov P., Ustyugova S., Illarionova A., Fushan A., Vinogradova T., Sverdlov E. (2003). The Human Genome Contains Many Types of Chimeric Retrogenes Generated through in Vivo RNA Recombination. Nucleic Acids Res..

[36] Lanciano S., Philippe C., Sarkar A., Pratella D., Domrane C., Doucet A.J., van Essen D., Saccani S., Ferry L., Defossez P.A. (2024). Locus-Level L1 DNA Methylation Profiling Reveals the Epigenetic and Transcriptional Interplay between L1s and Their Integration Sites. Cell Genom..

[37] Lee H.E., Ayarpadikannan S., Kim H.S. (2015). Role of Transposable Elements in Genomic Rearrangement, Evolution, Gene Regulation and Epigenetics in Primates. Genes Genet. Syst..

[38] Enriquez-Gasca R., Gould P.A., Tunbak H., Conde L., Herrero J., Chittka A., Beck C.R., Gifford R., Rowe H.M. (2023). Co-Option of Endogenous Retroviruses through Genetic Escape from TRIM28 Repression. Cell Rep..

[39] Enriquez-Gasca R., Gould P.A., Rowe H.M. (2020). Host Gene Regulation by Transposable Elements: The New, the Old and the Ugly. Viruses.

[40] Buttler C.A., Ramirez D., Dowell R.D., Chuong E.B. (2023). An Intronic LINE-1 Regulates IFNAR1 Expression in Human Immune Cells. Mob. DNA.

[41] Ng K.W., Attig J., Young G.R., Ottina E., Papamichos S.I., Kotsianidis I., Kassiotis G. (2019). Soluble PD-L1 Generated by Endogenous Retroelement Exaptation Is a Receptor Antagonist. eLife.

[42] Ahn H.W., Worman Z.F., Lechsinska A., Payer L.M., Wang T., Malik N., Li W., Burns K.H., Nath A., Levin H.L. (2023). Retrotransposon Insertions Associated with Risk of Neurologic and Psychiatric Diseases. EMBO Rep..

[43] Mavragani C.P., Sagalovskiy I., Guo Q., Nezos A., Kapsogeorgou E.K., Lu P., Liang Zhou J., Kirou K.A., Seshan S.V., Moutsopoulos H.M. (2016). Expression of Long Interspersed Nuclear Element 1 Retroelements and Induction of Type I Interferon in Patients With Systemic Autoimmune Disease. Arthritis Rheumatol..

[44] Aiken C., Rousso I. (2021). The HIV-1 Capsid and Reverse Transcription. Retrovirology.

[45] Warkocki Z. (2023). An Update on Post-Transcriptional Regulation of Retrotransposons. FEBS Lett..

[46] Protasova M.S., Andreeva T.V., Rogaev E.I. (2021). Factors Regulating the Activity of LINE1 Retrotransposons. Genes.

[47] Levin H.L., Moran J.V. (2011). Dynamic Interactions between Transposable Elements and Their Hosts. Nat. Rev. Genet..

[48] Hancks D.C., Kazazian H.H. (2016). Roles for Retrotransposon Insertions in Human Disease. Mob. DNA.

[49] Bodak M., Yu J., Ciaudo C. (2014). Regulation of LINE-1 in Mammals. Biomol. Concepts.

[50] Tie C.H., Fernandes L., Conde L., Robbez-Masson L., Sumner R.P., Peacock T., Rodriguez-Plata M.T., Mickute G., Gifford R., Towers G.J. (2018). KAP 1 Regulates Endogenous Retroviruses in Adult Human Cells and Contributes to Innate Immune Control. EMBO Rep..

[51] Kato M., Takemoto K., Shinkai Y. (2018). A Somatic Role for the Histone Methyltransferase Setdb1 in Endogenous Retrovirus Silencing. Nat. Commun..

[52] Greenberg M.V.C., Bourc’his D. (2019). The Diverse Roles of DNA Methylation in Mammalian Development and Disease. Nat. Rev. Mol. Cell Biol..

[53] Tie C.H., Rowe H.M. (2017). Epigenetic Control of Retrotransposons in Adult Tissues: Implications for Immune Regulation. Curr. Opin. Virol..

[54] Deniz Ö., Frost J.M., Branco M.R. (2019). Regulation of Transposable Elements by DNA Modifications. Nat. Rev. Genet..

[55] Barau J., Teissandier A., Zamudio N., Roy S., Nalesso V., Hérault Y., Guillou F., Bourc’his D. (2016). The DNA Methyltransferase DNMT3C Protects Male Germ Cells from Transposon Activity. Science.

[56] Pandiloski N., Horváth V., Karlsson O., Koutounidou S., Dorazehi F., Christoforidou G., Matas-Fuentes J., Gerdes P., Garza R., Jönsson M.E. (2024). DNA Methylation Governs the Sensitivity of Repeats to Restriction by the HUSH-MORC2 Corepressor. Nat. Commun..

[57] Sanchez-Luque F.J., Kempen M.J.H.C., Gerdes P., Vargas-Landin D.B., Richardson S.R., Troskie R.L., Jesuadian J.S., Cheetham S.W., Carreira P.E., Salvador-Palomeque C. (2019). LINE-1 Evasion of Epigenetic Repression in Humans. Mol. Cell.

[58] Schultz D.C., Ayyanathan K., Negorev D., Maul G.G., Rauscher F.J. (2002). SETDB1: A Novel KAP-1-Associated Histone H3, Lysine 9-Specific Methyltransferase That Contributes to HP1-Mediated Silencing of Euchromatic Genes by KRAB Zinc-Finger Proteins. Genes Dev..

[59] Collins P.L., Kyle K.E., Egawa T., Shinkai Y., Oltz E.M., Moran J.V. (2015). The Histone Methyltransferase SETDB1 Represses Endogenous and Exogenous Retroviruses in B Lymphocytes. Proc. Natl. Acad. Sci. USA.

[60] Cuellar L., Herzner A.M., Zhang X., Goyal Y., Watanabe C., Friedman B.A., Janakiraman V., Durinck S., Stinson J., Arnott D. (2017). Silencing of Retrotransposons by SET DB1 Inhibits the Interferon Response in Acute Myeloid Leukemia. J. Cell Biol..

[61] Ninova M., Tóth K.F., Aravin A.A. (2019). The Control of Gene Expression and Cell Identity by H3K9 Trimethylation. Development.

[62] Rowe H.M., Trono D. (2011). Dynamic Control of Endogenous Retroviruses during Development. Virology.

[63] Fukuda K., Okuda A., Yusa K., Shinkai Y. (2018). A CRISPR Knockout Screen Identifies SETDB1-Target Retroelement Silencing Factors in Embryonic Stem Cells. Genome Res..

[64] Ecco G., Cassano M., Kauzlaric A., Duc J., Coluccio A., Offner S., Imbeault M., Rowe H.M., Turelli P., Trono D. (2016). Transposable Elements and Their KRAB-ZFP Controllers Regulate Gene Expression in Adult Tissues. Dev. Cell.

[65] Griffin G.K., Wu J., Iracheta-Vellve A., Patti J.C., Hsu J., Davis T., Dele-Oni D., Du P.P., Halawi A.G., Ishizuka J.J. (2021). Epigenetic Silencing by SETDB1 Suppresses Tumour Intrinsic Immunogenicity. Nature.

[66] Sripathy S.P., Stevens J., Schultz D.C. (2006). The KAP1 Corepressor Functions To Coordinate the Assembly of De Novo HP1-Demarcated Microenvironments of Heterochromatin Required for KRAB Zinc Finger Protein-Mediated Transcriptional Repression. Mol. Cell. Biol..

[67] Imbeault M., Helleboid P.Y., Trono D. (2017). KRAB Zinc-Finger Proteins Contribute to the Evolution of Gene Regulatory Networks. Nature.

[68] Corsinotti A., Kapopoulou A., Gubelmann C., Imbeault M., Santoni de Sio F.R., Rowe H.M., Mouscaz Y., Deplancke B., Trono D. (2013). Global and Stage Specific Patterns of Krüppel-Associated-Box Zinc Finger Protein Gene Expression in Murine Early Embryonic Cells. PLoS ONE.

[69] Jacobs F.M.J., Greenberg D., Nguyen N., Haeussler M., Ewing A.D., Katzman S., Paten B., Salama S.R., Haussler D. (2014). An Evolutionary Arms Race between KRAB Zinc-Finger Genes ZNF91/93 and SVA/L1 Retrotransposons. Nature.

[70] Zhang T., Zhao W., Wirth C., Díaz-Gay M., Yin J., Cecati M., Marchegiani F., Hoang P.H., Leduc C., Baine M.K. (2025). Uncovering the Role of LINE-1 in the Evolution of Lung Adenocarcinoma. Nature.

[71] Bulut-Karslioglu A., DeLaRosa-Velázquez I.A., Ramirez F., Barenboim M., Onishi-Seebacher M., Arand J., Galán C., Winter G.E., Engist B., Gerle B. (2014). Suv39h-Dependent H3K9me3 Marks Intact Retrotransposons and Silences LINE Elements in Mouse Embryonic Stem Cells. Mol. Cell.

[72] Fritsch L., Robin P., Mathieu J.R.R., Souidi M., Hinaux H., Rougeulle C., Harel-Bellan A., Ameyar-Zazoua M., Ait-Si-Ali S. (2010). A Subset of the Histone H3 Lysine 9 Methyltransferases Suv39h1, G9a, GLP, and SETDB1 Participate in a Multimeric Complex. Mol. Cell.

[73] Tsusaka T., Kikuchi M., Shimazu T., Suzuki T., Sohtome Y., Akakabe M., Sodeoka M., Dohmae N., Umehara T., Shinkai Y. (2018). Tri-Methylation of ATF7IP by G9a/GLP Recruits the Chromodomain Protein. Epigenet. Chromatin.

[74] Timms R.T., Tchasovnikarova I.A., Antrobus R., Dougan G., Lehner P.J. (2016). ATF7IP-Mediated Stabilization of the Histone Methyltransferase SETDB1 Is Essential for Heterochromatin Formation by the HUSH Complex. Cell Rep..

[75] Tchasovnikarova I.A., Timms R.T., Matheson N.J., Wals K., Antrobus R., Göttgens B., Dougan G., Dawson M.A., Lehner P.J. (2015). Epigenetic Silencing by the HUSH Complex Mediates Position-Effect Variegation in Human Cells. Science.

[76] Liu N., Lee C.H., Swigut T., Grow E., Gu B., Bassik M.C., Wysocka J. (2017). Selective Silencing of Euchromatic L1s Revealed by Genome-Wide Screens for L1 Regulators. Nature.

[77] Robbez-Masson L., Tie C.H.C., Conde L., Tunbak H., Husovsky C., Tchasovnikarova I.A., Timms R.T., Herrero J., Lehner P.J., Rowe H.M. (2018). The Hush Complex Cooperates with Trim28 to Repress Young Retrotransposons and New Genes. Genome Res..

[78] Lehner P.J. (2025). Silencing by the HUSH Epigenetic Transcriptional Repressor Complex. Annu. Rev. Biochem..

[79] Seczynska M., Bloor S., Cuesta S.M., Lehner P.J. (2021). Genome Surveillance by HUSH-Mediated Silencing of Intronless Mobile Elements. Nature.

[80] Tunbak H., Enriquez-Gasca R., Tie C.H.C., Gould P.A., Mlcochova P., Gupta R.K., Fernandes L., Holt J., van der Veen A.G., Giampazolias E. (2020). The HUSH Complex Is a Gatekeeper of Type I Interferon through Epigenetic Regulation of LINE-1s. Nat. Commun..

[81] Douse C.H., Tchasovnikarova I.A., Timms R.T., Protasio A.V., Seczynska M., Prigozhin D.M., Albecka A., Wagstaff J., Williamson J.C., Freund S.M.V. (2020). TASOR Is a Pseudo-PARP That Directs HUSH Complex Assembly and Epigenetic Transposon Control. Nat. Commun..

[82] Bloor S., Wit N., Lehner P.J. (2025). RNA Binding by Periphilin Plays an Essential Role in Initiating Silencing by the HUSH Complex. Nucleic Acids Res..

[83] Prigozhin D.M., Douse C.H., Farleigh L.E., Albecka A., Tchasovnikarova I.A., Timms R.T., Oda S.I., Adolf F., Freund S.M.V., Maslen S. (2020). Periphilin Self-Association Underpins Epigenetic Silencing by the HUSH Complex. Nucleic Acids Res..

[84] Müller I., Moroni A.S., Shlyueva D., Sahadevan S., Schoof E.M., Radzisheuskaya A., Højfeldt J.W., Tatar T., Koche R.P., Huang C. (2021). MPP8 Is Essential for Sustaining Self-Renewal of Ground-State Pluripotent Stem Cells. Nat. Commun..

[85] Tchasovnikarova I.A., Timms R.T., Douse C.H., Roberts R.C., Dougan G., Kingston R.E., Modis Y., Lehner P.J. (2017). Hyperactivation of HUSH Complex Function by Charcot–Marie–Tooth Disease Mutation in MORC2. Nat. Genet..

[86] Douse C.H., Bloor S., Liu Y., Shamin M., Tchasovnikarova I.A., Timms R.T., Lehner P.J., Modis Y. (2018). Neuropathic MORC2 Mutations Perturb GHKL ATPase Dimerization Dynamics and Epigenetic Silencing by Multiple Structural Mechanisms. Nat. Commun..

[87] Moissiard G., Cokus S.J., Cary J., Feng S., Billi A.C., Stroud H., Husmann D., Zhan Y., Lajoie B.R., McCord R.P. (2012). MORC Family ATPases Required for Heterochromatin Condensation and Gene Silencing. Science.

[88] Garland W., Müller I., Wu M., Schmid M., Imamura K., Rib L., Sandelin A., Helin K., Jensen T.H. (2022). Chromatin Modifier HUSH Co-Operates with RNA Decay Factor NEXT to Restrict Transposable Element Expression. Mol. Cell.

[89] Spencley A.L., Bar S., Swigut T., Flynn R.A., Lee C.H., Chen L.F., Bassik M.C., Wysocka J. (2023). Co-Transcriptional Genome Surveillance by HUSH Is Coupled to Termination Machinery. Mol. Cell.

[90] Danac J.M.C., Matthews R.E., Gungi A., Qin C., Parsons H., Antrobus R., Timms R.T., Tchasovnikarova I.A. (2024). Competition between Two HUSH Complexes Orchestrates the Immune Response to Retroelement Invasion. Mol. Cell.

[91] Jensvold Z.D., Flood J.R., Christenson A.E., Lewis P.W. (2024). Interplay between Two Paralogous Human Silencing Hub (HuSH) Complexes in Regulating LINE-1 Element Silencing. Nat. Commun..

[92] Holt J.H., Enriquez-Gasca R., Wilson R.P., Bochukova E.G., Maillard P.V., Rowe H.M. (2026). Epigenetic Lockdown of Type I Interferon Sensing and Signalling in Human Pluripotent Cells. Nat. Commun..

[93] Adami A., Garza R., Gerdes P., Johansson P.A., Dorazehi F., Koutounidou S., Castilla-Vallmanya L., Atacho D.A.M., Sharma Y., Johansson J.G. (2025). LINE-1 Retrotransposons Mediate Cis-Acting Transcriptional Control in Human Pluripotent Stem Cells and Regulate Early Brain Development. Cell Genom..

[94] Chang Y., Sun L., Kokura K., Horton J.R., Fukuda M., Espejo A., Izumi V., Koomen J.M., Bedford M.T., Zhang X. (2011). MPP8 Mediates the Interactions between DNA Methyltransferase Dnmt3a and H3K9 Methyltransferase GLP/G9a. Nat. Commun..

[95] De Cecco M., Ito T., Petrashen A.P., Elias A.E., Skvir N.J., Criscione S.W., Caligiana A., Brocculi G., Adney E.M., Boeke J.D. (2019). L1 Drives IFN in Senescent Cells and Promotes Age-Associated Inflammation. Nature.

[96] Tang H., Yang J., Xu J., Zhang W., Geng A., Jiang Y., Mao Z. (2024). The Transcription Factor PAX5 Activates Human LINE1 Retrotransposons to Induce Cellular Senescence. EMBO Rep..

[97] Kong Y., Rose C.M., Cass A.A., Williams A.G., Darwish M., Lianoglou S., Haverty P.M., Tong A.J., Blanchette C., Albert M.L. (2019). Transposable Element Expression in Tumors Is Associated with Immune Infiltration and Increased Antigenicity. Nat. Commun..

[98] Szpakowski S., Sun X., Lage J.M., Dyer A., Rubinstein J., Kowalski D., Sasaki C., Costa J., Lizardi P.M. (2009). Loss of Epigenetic Silencing in Tumors Preferentially Affects Primate-Specific Retroelements. Gene.

[99] Baba Y., Yagi T., Sawayama H., Hiyoshi Y., Ishimoto T., Iwatsuki M., Miyamoto Y., Yoshida N., Baba H. (2018). Long Interspersed Element-1 Methylation Level as a Prognostic Biomarker in Gastrointestinal Cancers. Digestion.

[100] Pezic D., Manakov S.A., Sachidanandam R., Aravin A.A. (2014). PiRNA Pathway Targets Active LINE1 Elements to Establish the Repressive H3K9me3 Mark in Germ Cells. Genes Dev..

[101] Arora R., Bodak M., Penouty L., Hackman C., Ciaudo C. (2022). Sequestration of LINE-1 in Cytosolic Aggregates by MOV10 Restricts Retrotransposition. EMBO Rep..

[102] Luqman-Fatah A., Watanabe Y., Uno K., Ishikawa F., Moran J.V., Miyoshi T. (2023). The Interferon Stimulated Gene-Encoded Protein HELZ2 Inhibits Human LINE-1 Retrotransposition and LINE-1 RNA-Mediated Type I Interferon Induction. Nat. Commun..

[103] Mita P., Sun X., Fenyö D., Kahler D.J., Li D., Agmon N., Wudzinska A., Keegan S., Bader J.S., Yun C. (2020). BRCA1 and S Phase DNA Repair Pathways Restrict LINE-1 Retrotransposition in Human Cells. Nat. Struct. Mol. Biol..

[104] Oksuz O., Chu C., Arisdakessian C., Diao L., Zaller D., Long K.K., Keilhack H., Knutson S. (2025). Identification of Novel Regulators of LINE-1 Expression via CRISPR/Cas9 Screening. Mob. DNA.

[105] Li X., Bie L., Wang Y., Hong Y., Zhou Z., Fan Y., Yan X., Tao Y., Huang C., Zhang Y. (2024). LINE-1 Transcription Activates Long-Range Gene Expression. Nat. Genet..

[106] Briscoe J., Guschin D., Rogers N.C., Watling D., Müller M., Horn F., Heinrich P., Stark G.R., Kerr I.M. (1996). JAKs, STATs and Signal Transduction in Response to the Interferons and Other Cytokines. Philos. Trans. R. Soc. Lond. B Biol. Sci..

[107] Schneider W.M., Chevillotte M.D., Rice C.M. (2014). Interferon-Stimulated Genes: A Complex Web of Host Defenses. Annu. Rev. Immunol..

[108] Zhao X., Zhao Y., Du J., Gao P., Zhao K. (2021). The Interplay Among HIV, LINE-1, and the Interferon Signaling System. Front. Immunol..

[109] Mathavarajah S., Dellaire G. (2024). LINE-1: An Emerging Initiator of CGAS-STING Signalling and Inflammation That Is Dysregulated in Disease. Biochem. Cell Biol..

[110] Chen Y.G., Hur S. (2021). Cellular Origins of DsRNA, Their Recognition and Consequences. Nat. Rev. Mol. Cell Biol..

[111] Hur S. (2019). Double-Stranded RNA Sensors and Modulators in Innate Immunity. Annu. Rev. Immunol..

[112] Stok J.E., Vega Quiroz M.E., van der Veen A.G. (2020). Self RNA Sensing by RIG-I–like Receptors in Viral Infection and Sterile Inflammation. J. Immunol..

[113] Rehwinkel J., Gack M.U. (2020). RIG-I-like Receptors: Their Regulation and Roles in RNA Sensing. Nat. Rev. Immunol..

[114] Yoneyama M., Kikuchi M., Natsukawa T., Shinobu N., Imaizumi T., Miyagishi M., Taira K., Akira S., Fujita T. (2004). The RNA Helicase RIG-I Has an Essential Function in Double-Stranded RNA-Induced Innate Antiviral Responses. Nat. Immunol..

[115] Roulois D., Loo Yau H., Singhania R., Wang Y., Danesh A., Shen S.Y., Han H., Liang G., Jones P.A., Pugh T.J. (2015). DNA-Demethylating Agents Target Colorectal Cancer Cells by Inducing Viral Mimicry by Endogenous Transcripts. Cell.

[116] Chiappinelli K.B., Strissel P.L., Desrichard A., Li H., Henke C., Akman B., Hein A., Rote N.S., Cope L.M., Snyder A. (2015). Inhibiting DNA Methylation Causes an Interferon Response in Cancer via DsRNA Including Endogenous Retroviruses. Cell.

[117] Luecke S., Holleufer A., Christensen M.H., Jønsson K.L., Boni G.A., Sørensen L.K., Johannsen M., Jakobsen M.R., Hartmann R., Paludan S.R. (2017). CGAS Is Activated by DNA in a Length-dependent Manner. EMBO Rep..

[118] Liu S., Cai X., Wu J., Cong Q., Chen X., Li T., Du F., Ren J., Wu Y.T., Grishin N.V. (2015). Phosphorylation of Innate Immune Adaptor Proteins MAVS, STING, and TRIF Induces IRF3 Activation. Science.

[119] Sun L., Wu J., Du F., Chen X., Chen Z.J. (2013). Cyclic GMP-AMP Synthase Is a Cytosolic DNA Sensor That Activates the Type I Interferon Pathway. Science.

[120] Zhang X., Bai X.-C., Chen Z.J. (2020). Structures and Mechanisms in the CGAS-STING Innate Immunity Pathway. Immunity.

[121] Mankan A.K., Schmidt T., Chauhan D., Goldeck M., Höning K., Gaidt M., Kubarenko A.V., Andreeva L., Hopfner K., Hornung V. (2014). Cytosolic RNA:DNA Hybrids Activate the CGAS –STING Axis. EMBO J..

[122] Lee K., Ku J., Ku D., Kim Y. (2024). Inverted Alu Repeats: Friends or Foes in the Human Transcriptome. Exp. Mol. Med..

[123] Kim S., Ku Y., Ku J., Kim Y. (2019). Evidence of Aberrant Immune Response by Endogenous Double-Stranded RNAs: Attack from Within. Bioessays.

[124] Ahmad S., Mu X., Yang F., Greenwald E., Park J.W., Jacob E., Zhang C.Z., Hur S. (2018). Breaching Self-Tolerance to Alu Duplex RNA Underlies MDA5-Mediated Inflammation. Cell.

[125] Aune T.M., Tossberg J.T., Heinrich R.M., Porter K.P., Crooke P.S. (2022). Alu RNA Structural Features Modulate Immune Cell Activation and A-to-I Editing of Alu RNAs Is Diminished in Human Inflammatory Bowel Disease. Front. Immunol..

[126] Kim Y., Lee J.H., Park J.E., Cho J., Yi H., Kim V.N. (2014). PKR Is Activated by Cellular DsRNAs during Mitosis and Acts as a Mitotic Regulator. Genes Dev..

[127] Chung J., Lee Y., Yoon J., Kim Y. (2025). Deciphering the Multifaceted Role of Double-Stranded RNA Sensor Protein Kinase R: Pathophysiological Function beyond the Antiviral Response. RNA Biol..

[128] de Reuver R., Maelfait J. (2023). Novel Insights into Double-Stranded RNA-Mediated Immunopathology. Nat. Rev. Immunol..

[129] Rice G.I., Kasher P.R., Forte G.M.A., Mannion N.M., Greenwood S.M., Szynkiewicz M., Dickerson J.E., Bhaskar S.S., Zampini M., Briggs T.A. (2012). Mutations in ADAR1 Cause Aicardi-Goutières Syndrome Associated with a Type I Interferon Signature. Nat. Genet..

[130] Rice G.I., Del Toro Duany Y., Jenkinson E.M., Forte G.M.A., Anderson B.H., Ariaudo G., Bader-Meunier B., Baildam E.M., Battini R., Beresford M.W. (2014). Gain-of-Function Mutations in IFIH1 Cause a Spectrum of Human Disease Phenotypes Associated with Upregulated Type I Interferon Signaling. Nat. Genet..

[131] Yu Q., Herrero del Valle A., Singh R., Modis Y. (2021). MDA5 Disease Variant M854K Prevents ATP-Dependent Structural Discrimination of Viral and Cellular RNA. Nat. Commun..

[132] Thomas C.A., Tejwani L., Trujillo C.A., Negraes P.D., Herai R.H., Mesci P., Macia A., Crow Y.J., Muotri A.R. (2017). Modeling of TREX1-Dependent Autoimmune Disease Using Human Stem Cells Highlights L1 Accumulation as a Source of Neuroinflammation. Cell Stem Cell.

[133] Gulen M.F., Samson N., Keller A., Schwabenland M., Liu C., Glück S., Thacker V.V., Favre L., Mangeat B., Kroese L.J. (2023). CGAS–STING Drives Ageing-Related Inflammation and Neurodegeneration. Nature.

[134] Witteveldt J., Liu Z., Ariza-Cosano A., Ramirez C., Walters J.L., Marchante P.G., Maas L., Ivens A., Tebaldi T., Heras S.R. (2025). Double Stranded RNA Sensing Drives Interferon Silencing in Early Development. bioRxiv.

[135] Mangiavacchi A., Morelli G., Reppe S., Saera-Vila A., Liu P., Eggerschwiler B., Zhang H., Bensaddek D., Casanova E.A., Medina Gomez C. (2024). LINE-1 RNA Triggers Matrix Formation in Bone Cells via a PKR-Mediated Inflammatory Response. EMBO J..

[136] Zhen Z., Chen Y., Wang H., Tang H., Zhang H., Liu H., Jiang Y., Mao Z. (2023). Nuclear CGAS Restricts L1 Retrotransposition by Promoting TRIM41-Mediated ORF2p Ubiquitination and Degradation. Nat. Commun..

[137] Yan J., Zhao Y., Du J., Wang Y., Wang S., Wang Q., Zhao X., Xu W., Zhao K. (2022). RNA Sensor MDA5 Suppresses LINE-1 Retrotransposition by Regulating the Promoter Activity of LINE-1 5′-UTR. Mob. DNA.

[138] Orecchini E., Doria M., Antonioni A., Galardi S., Ciafré S.A., Frassinelli L., Mancone C., Montaldo C., Tripodi M., Michienzi A. (2017). ADAR1 Restricts LINE-1 Retrotransposition. Nucleic Acids Res..

[139] Stetson D.B., Ko J.S., Heidmann T., Medzhitov R. (2008). Trex1 Prevents Cell-Intrinsic Initiation of Autoimmunity. Cell.

[140] Gázquez-Gutiérrez A., Chin P., Peris G., Gordon K., Marchante P.G., Witteveldt J., Tristán-Ramos P., Rouvière J.O., Knol L.I., Ivens A. (2026). Control of Retrotransposon-Driven Activation of the Interferon Response by the Double-Stranded RNA Binding Protein DGCR8. Nucleic Acids Res..

[141] Guan Y., Gao H., Leu N.A., Vourekas A., Alexiou P., Maragkakis M., Kang Z., Mourelatos Z., Liang G., Wang P.J. (2023). The MOV10 RNA Helicase Is a Dosage-Dependent Host Restriction Factor for LINE1 Retrotransposition in Mice. PLoS Genet..

[142] Liu Q., Liu Y., Mao Y., Yi D., Li Q., Ding J., Guo S., Zhang Y., Wang J., Zhao J. (2025). Maximal Inhibitory Effect of MOV10 on LINE-1 Retrotransposition Requires Both the MOV10/LINE-1 Association and Granule Formation. PLoS Genet..

[143] Zhang A., Dong B., Doucet A.J., Moldovan J.B., Moran J.V., Silverman R.H. (2013). RNase L Restricts the Mobility of Engineered Retrotransposons in Cultured Human Cells. Nucleic Acids Res..

[144] You E., Patel B.K., Rojas A.S., Sun S., Danaher P., Ho N.I., Phillips I.E., Raabe M.J., Song Y., Xu K.H. (2025). LINE-1 ORF1p Mimics Viral Innate Immune Evasion Mechanisms in Pancreatic Ductal Adenocarcinoma. Cancer Discov..

[145] Lau A., Gray E.E., Brunette R.L., Stetson D.B. (2015). DNA Tumor Virus Oncogenes Antagonize the CGAS-STING DNA-Sensing Pathway. Science.

[146] Sun S., You E., Hong J., Hoyos D., Del Priore I., Tsanov K.M., Mattagajasingh O., Di Gioacchino A., Marhon S.A., Chacon-Barahona J. (2024). Cancer Cells Restrict Immunogenicity of Retrotransposon Expression via Distinct Mechanisms. Immunity.

[147] Yang R., Yu S., Xu T., Zhang J., Wu S. (2022). Emerging Role of RNA Sensors in Tumor Microenvironment and Immunotherapy. J. Hematol. Oncol..

[148] Mehdipour P., Marhon S.A., Ettayebi I., Chakravarthy A., Hosseini A., Wang Y., de Castro F.A., Loo Yau H., Ishak C., Abelson S. (2020). Epigenetic Therapy Induces Transcription of Inverted SINEs and ADAR1 Dependency. Nature.

[149] Larouche J.D., Trofimov A., Hesnard L., Ehx G., Zhao Q., Vincent K., Durette C., Gendron P., Laverdure J.P., Bonneil É. (2020). Widespread and Tissue-Specific Expression of Endogenous Retroelements in Human Somatic Tissues. Genome Med..

[150] Larouche J.-D., Laumont C.M., Trofimov A., Vincent K., Hesnard L., Brochu S., Côté C., Humeau J., Bonneil É., Lanoix J. (2024). Transposable Elements Regulate Thymus Development and Function. eLife.

[151] Carter J.A., Strömich L., Peacey M., Chapin S.R., Velten L., Steinmetz L.M., Brors B., Pinto S., Meyer H.V. (2022). Transcriptomic Diversity in Human Medullary Thymic Epithelial Cells. Nat. Commun..

[152] Chapman N.M., Boothby M.R., Chi H. (2020). Metabolic Coordination of T Cell Quiescence and Activation. Nat. Rev. Immunol..

[153] Marasca F., Sinha S., Vadalà R., Polimeni B., Ranzani V., Paraboschi E.M., Burattin F.V., Ghilotti M., Crosti M., Negri M.L. (2022). LINE1 Are Spliced in Non-Canonical Transcript Variants to Regulate T Cell Quiescence and Exhaustion. Nat. Genet..

[154] Burattin F.V., Vadalà R., Panepuccia M., Ranzani V., Crosti M., Colombo F.A., Ruberti C., Erba E., Prati D., Nittoli T. (2024). LINE1 Modulate Human T Cell Function by Regulating Protein Synthesis during the Life Span. Sci. Adv..

[155] Wells A.C., Lima-Junior D.S., Link V.M., Smelkinson M., Krishnamurthy S.R., Chi L., Segrist E., Rivera C.A., Teijeiro A., Bouladoux N. (2024). Adaptive Immunity to Retroelements Promotes Barrier Integrity. bioRxiv.

[156] Libera L., Vanoli A., Sahnane N., Adnan M., Guerini C., Arpa G., Bianchi P.I., Lenti M.V., Corazza G.R., La Rosa S. (2025). LINE-1 Hypomethylation Characterizes the Inflammatory Response in Coeliac Disease Associated-Intestinal Mucosa and Small Bowel Adenocarcinomas. J. Pathol..

[157] Benihoud K., Bonardelle D., Soual-Hoebeke E., Durand-Gasselin I., Emilie D., Kiger N., Bobé P. (2002). Unusual Expression of LINE-1 Transposable Element in the MRL Autoimmune Lymphoproliferative Syndrome-Prone Strain. Oncogene.

[158] Shah N.M., Jang H.J., Liang Y., Maeng J.H., Tzeng S.C., Wu A., Basri N.L., Qu X., Fan C., Li A. (2023). Pan-Cancer Analysis Identifies Tumor-Specific Antigens Derived from Transposable Elements. Nat. Genet..

[159] Bonaventura P., Alcazer V., Mutez V., Tonon L., Martin J., Chuvin N., Michel E., Boulos R.E., Estornes Y., Valladeau-Guilemond J. (2022). Identification of Shared Tumor Epitopes from Endogenous Retroviruses Inducing High-Avidity Cytotoxic T Cells for Cancer Immunotherapy. Sci. Adv..

[160] Oka M., Xu L., Suzuki T., Yoshikawa T., Sakamoto H., Uemura H., Yoshizawa A.C., Suzuki Y., Nakatsura T., Ishihama Y. (2021). Aberrant Splicing Isoforms Detected by Full-Length Transcriptome Sequencing as Transcripts of Potential Neoantigens in Non-Small Cell Lung Cancer. Genome Biol..

[161] McKerrow W., Snyder M., Chu C., Kelly D., Keilhack H., Diao L. (2025). LINE-1 ORF1p Is a Shared and Immunogenic Antigen in Cancer. bioRxiv.

[162] Lee B., Park J., Voshall A., Maury E., Kang Y., Kim Y.J., Lee J.-Y., Shim H.-R., Kim H.-J., Lee J.-W. (2023). Pan-Cancer Analysis Reveals Multifaceted Roles of Retrotransposon-Fusion RNAs. bioRxiv.

[163] Kim S., Shin W., Lee Y.M., Mun S., Han K. (2020). Differential Expressions of L1-Chimeric Transcripts in Normal and Matched-Cancer Tissues. Anal. Biochem..

[164] Li Y., Liu Y., Xie Y., Wang Y., Wang J., Wang H., Xia L., Xie D. (2025). Long-Read RNA Sequencing Enables Full-Length Chimeric Transcript Annotation of Transposable Elements in Lung Adenocarcinoma. BMC Cancer.

[165] Wolff E.M., Byun H.M., Han H.F., Sharma S., Nichols P.W., Siegmund K.D., Yang A.S., Jones P.A., Liang G. (2010). Hypomethylation of a LINE-1 Promoter Activates an Alternate Transcript of the MET Oncogene in Bladders with Cancer. PLoS Genet..

[166] Miglio U., Berrino E., Panero M., Ferrero G., Coscujuela Tarrero L., Miano V., Dell’Aglio C., Sarotto I., Annaratone L., Marchiò C. (2018). The Expression of LINE1-MET Chimeric Transcript Identifies a Subgroup of Aggressive Breast Cancers. Int. J. Cancer.

[167] Burbage M., Rocañín-Arjó A., Baudon B., Arribas Y.A., Merlotti A., Rookhuizen D.C., Heurtebise-Chrétien S., Ye M., Houy A., Burgdorf N. (2023). Epigenetically Controlled Tumor Antigens Derived from Splice Junctions between Exons and Transposable Elements. Sci. Immunol..

[168] Vafadar-Isfahani N., Parr C., McMillan L.E., Sanner J., Yeo Z., Saddington S., Peacock O., Cruickshanks H.A., Meehan R.R., Lund J.N. (2017). Decoupling of DNA Methylation and Activity of Intergenic LINE-1 Promoters in Colorectal Cancer. Epigenetics.

[169] Chong C., Müller M., Pak H.S., Harnett D., Huber F., Grun D., Leleu M., Auger A., Arnaud M., Stevenson B.J. (2020). Integrated Proteogenomic Deep Sequencing and Analytics Accurately Identify Non-Canonical Peptides in Tumor Immunopeptidomes. Nat. Commun..

[170] Burns K.H. (2017). Transposable Elements in Cancer. Nat. Rev. Cancer.

[171] Mendez-Dorantes C., Burns K.H. (2023). LINE-1 Retrotransposition and Its Deregulation in Cancers: Implications for Therapeutic Opportunities. Genes Dev..

